# Evaluating Population-Level Interventions and Exposures for Suicide Prevention

**DOI:** 10.1027/0227-5910/a000961

**Published:** 2024-05-21

**Authors:** Matthew J. Spittal, David Gunnell, Mark Sinyor, Angela Clapperton, Leo Roberts, Jane Pirkis, Thomas Niederkrotenthaler

**Affiliations:** ^1^ Centre for Mental Health and Community Wellbeing, Melbourne School of Population and Global Health, The University of Melbourne, VIC, Australia; ^2^ National Institute of Health and Care Research Biomedical Research Centre, University Hospitals Bristol and Weston NHS Foundation Trust, University of Bristol, UK; ^3^ Department of Psychiatry, Sunnybrook Health Sciences Centre, Toronto, Canada; ^4^ Department of Psychiatry, University of Toronto, Canada; ^5^ Unit Suicide Research and Mental Health Promotion, Department of Social and Preventive Medicine, Center for Public Health, Medical University of Vienna, Austria

**Keywords:** policy evaluation, pre–post design, difference-in-difference design, time series analysis, interrupted time series analysis

## Abstract

**Abstract:** Evaluations of interventions targeting the population level are an essential component of the policy development cycle. Pre–post designs are widespread in suicide prevention research but have several significant limitations. To inform future evaluations, our aim is to explore the three most frequently used approaches for assessing the association between population-level interventions or exposures and suicide – the pre–post design, the difference-in-difference design, and Poisson regression approaches. The pre–post design and the difference-in-difference design will only produce unbiased estimates of an association if there are no underlying time trends in the data and there is no additional confounding from other sources. Poisson regression approaches with covariates for time can control for underlying time trends as well as the effects of other confounding factors. Our recommendation is that the default position should be to model the effects of population-level interventions or exposures using regression methods that account for time effects. The other designs should be seen as fall-back positions when insufficient data are available to use methods that control for time effects.

Many interventions in suicide prevention are targeted at the population level, for instance, withdrawing access to a widespread substance to prevent fatal overdoses (Hawton et al., 2009). Similarly many exposures operate at the population level, such as the exposure to poor media reporting about suicide (Niederkrotenthaler et al., 2020). In both cases, policy interest often lies in understanding the association between the specific intervention or exposure and suicidal behavior. Yet, because these interventions and exposures operate at the population level, as opposed to the individual level, it is a challenge to accurately measure the association in a way that does not introduce bias or confounding. Failure to account for bias or confounding could result in valuable interventions not being recognized and, equally, harmful exposures not being identified.

Evaluations of interventions targeting the population level are an essential component of the policy development cycle; therefore, an adequate methodological approach to evaluation is necessary to inform policy. There are three main approaches that are used in the field of suicide prevention to evaluate population-level interventions and exposures. These are the pre–post design, the difference-in-difference design and Poisson regression approaches. Our aims are to (1) describe each design, giving guidance about the interpretation of key parameters and highlighting each design’s strengths and limitations; (2) describe the interplay between study design and confounding when measuring population-level associations; (3) show how different study designs are linked by a common analytic approach; and (4) make recommendations about which design to use. Common across all three designs is the use of Poisson regression to model suicide counts. We focus on Poisson regression because it naturally models the counts of events in time, and it can model zero counts. In addition, effect sizes from a Poisson regression model can be interpreted as rate ratios. This approach can be applied to any outcome variable that is based on counts of events (e.g., number of traffic accidents, number of homicides). While it is possible to use linear regression to fit the same models, this approach cannot be used when zero counts are observed over a period of time. This can be problematic for pre–post designs and difference-in-difference designs.

We illustrate each of these designs with examples from the literature. We have chosen these examples because they are well conducted studies that have undertaken appropriate analyses. We have also developed an appendix that contains examples of how to set up the data in a way that is appropriate for each design, example code to undertake the analyses in Stata and R, and commentary aiding the interpretation of the results. We recommend reading the appendix alongside this paper. It is available as Electronic Supplementary Material 1 [ESM 1].

## The Pre–Post Design

In pre–post studies, a comparison is made between the number of suicides before and after an intervention or exposure, adjusting for time under observation. A variation on this approach is to compare a specific period of time in a year (e.g., April–June 2024) to the same periods in one or more preceding years, thereby controlling for seasonal fluctuations in suicide which have been observed in some studies (Galvão et al., 2018; Yu et al., 2020). This design is illustrated by a study of rail suicides following the death and subsequent media reporting of a celebrity by the same method (Ladwig et al., 2012). One hundred twenty-one suicides were observed in the 28 days following the celebrity’s suicide (the exposed period), and this was compared with 53 suicides were observed in the 28 days preceding it (the unexposed period). These data are available as ESM 2.

If $y_{e}$ and $y_{u}$ are the number of events in the exposed and unexposed periods (denoted by the e and u subscripts) and $t_{e}$ and $t_{u}$ are the time at risk in the same periods, then the rate ratio, RR, can be calculated using the formula,

$$\text{RR}=\frac{y_{e}/t_{e}}{y_{u}/t_{u}\;}.$$

The numerator, $y_{e}/t_{e}$, is the rate in the exposed period, and the denominator, $y_{u}/t_{u}$, is the rate in the unexposed period. A rate ratio less than 1 is interpreted as a reduction in the rate in the exposed period compared with the rate in the unexposed period. A rate ratio greater than 1 indicates an increase in the rate, and a rate ratio of 1 indicates no change.

This rate ratio can also be calculated using Poisson regression. This approach requires there to be two lines of data in the dataset with variables for number of suicides *y_i_*, the exposure period (coded 0 for the unexposed period and 1 for the exposed period) and the time at risk, $t_{i}$. The equation to fit this model is:

$$\log\left(y_{i}\right)=\beta_{0}+\beta_{1}\times {\text{exposure}}_{i}+\log\left(t_{i}\right).$$

The exponential of $\beta_{0}$, that is $e^{\beta_{0}},$ is interpreted as the rate in the unexposed period and the exponential of $\beta_{0}+\beta_{1}$, that is $e^{\beta_{0}+\beta_{1}},$ is the rate in the exposed period. Therefore ${e^{\beta_{0}+\beta_{1}}/e^{\beta_{0}}=e}^{\beta_{1}}=\text{RR}$, the change in the suicide rate between the exposed and unexposed periods.

Returning to the rail suicide example, the rate in the exposed period was 121/28 = 4.32 suicides per day and the rate in unexposed period was 53/28 = 1.89 suicides per day. The RR was therefore 4.32/1.89 = 2.28 and is interpreted as the number of suicides being 2.28 times higher in the exposed period compared to the unexposed period. When fit with Poisson regression, the RR was the same value as above and the 95% confidence interval (CI) was 1.65 to 3.15.

The pre–post approach is appealing because of its simplicity but makes two strong assumptions. One assumption is that there is no underlying time trend in the data. If there is a trend – for example, an increase in the suicide rate over time – then any observed change between the two periods could be partially or wholly due to this and not the exposure. The second assumption is that there are no other confounding factors. If confounding factors co-occur alongside the exposure, then any observed change might also be due to this.

The pre–post study’s other major limitation is that the design potentially has low statistical power. This is because the power to detect differences between the unexposed and exposed periods comes from the number of events in each period. All else being equal, a study with more suicides will have greater power than a study with fewer suicides. If there is only a small window in which to observe suicides, then there may not be enough observations to detect a difference between the two periods. Power could be increased by lengthening the observation time, but this increases the risk of bias since time trends and the occurrence of other events are likely to further influence the results and induce bias if the exposure effects do not last that long.

## The Difference-in-Difference Design

One solution to the problems inherent in the pre–post design is to instead use a difference-in-difference design. If the pre–post compares the number of suicides before and after an exposure, then the difference-in-difference design does this with the inclusion of a control group. The control might be another location or the same periods in the year prior.

One example of this design is a study of jumping suicides at the Bloor Street Viaduct (Sinyor et al., 2017) where a barrier was installed to prevent suicides. Prior to installation, there were 9.5 suicides per year; afterwards there were 0.1 suicides per year. The comparison group was jumping suicides from other bridges in Toronto. The rate at other bridges was 10.1 suicides per year prior to installation of the barrier at the viaduct and 11.0 per year after installation. Therefore, the pre–post rate ratio was 0.01 (95% CI 0.01–0.05) at the viaduct and 1.09 (95% CI 0.84–1.42) at the other bridges. These data are available as ESM 3. Similarly, Clapperton and colleagues studied the removal of level crossings (where the road intersects with rail tracks) on the incidence of rail suicide in Melbourne, Australia (Clapperton et al., 2022). They gathered data from 41 intervention sites where the level crossings had been removed and 41 control sites where the level crossing was yet to be removed. Each intervention site was matched with a control site, and this matching was used to anchor the pre–post comparison in each site on the date of the level crossing removal. In the intervention sites, the pre–post ratio was 0.32 (95% CI 0.11–0.74) and in the control sites it was 0.88 (95% CI 0.47–1.56). A notable strength of this study was the use of multiple intervention and control sites.

The difference-in-difference design tests whether the rate ratio for the period effect in the intervention arm is different from the period effect in the control arm. The RR is defined as:

$$\text{RR}=\frac{{\text{RR}}_{e}}{{\text{RR}}_{c}}$$

where e is the intervention arm and c is the control arm. A RR of 1 implies that there is no difference in suicides between ${\text{RR}}_{e}$ and ${\text{RR}}_{c}$, whereas a RR greater than 1 indicates an increase in suicides in the intervention arm and a RR less than 1 indicates a decrease. Few studies in the suicide prevention field formally test whether RR is different from the null value. Rather, they typically rely on informal evidence such as observing a significant change in the intervention arm but no significant change in the control arm. However, this can be done by extending the Poisson regression model to include terms for arm (control, intervention), period (unexposed, exposed), and a term for their interaction. This model should also a time variable capturing the duration of observation periods. Specifically,

$$\begin{array}{c} \log\left(y_{i}\right)=\beta_{0}+\beta_{1\;}\times {\text{intervention}\;\text{arm}}_{i}+\beta_{2}\times {\text{exposure}}_{i}+\\ \beta_{3}\times {\text{intervention}\;\text{arm}}_{i}\times {\text{exposure}}_{i}+\log\left(\text{tim}e_{i}\right).\; \end{array}$$

The key parameter is $\beta_{3}$ since its exponential, $e^{\beta_{3}}$ represents the RR. Applied to the Bloor Street Viaduct study, $\text{RR}=\frac{0.01}{1.09}=0.009$ with 95% CI 0.001 to 0.063. In the level crossing removal study, $\text{RR}=\frac{0.32}{0.88}=0.36$ with 95% CI 0.13 to 0.97.

The main assumption with the difference-in-difference design is that the underlying time trend in each arm is the same, differing by a fixed amount (Wing et al., 2018). This is known as parallel trends assumption. This assumption breaks when each arm has its own time trend or when there are other unmeasured confounding factors influencing the arms in different ways. A more general issue with the difference-in-difference design concerns the selection of the control group to minimize bias. In suicide prevention research, the control arm is often selected from prior years. Occasionally, data from subsequent years are included in the control arm too. This makes sense if the underlying suicide rate follows a similar pattern in each year but less so when the rate is unpredictable, has a nonlinear trend, or if the exposure has a long-term impact. Complications arise when a known confounding event has occurred in the control arm, necessitating the selection of a replacement control period. Some studies attempt to resolve this issue by selecting data from the same period as the intervention arm but at a different location. This works well if there is no possibility of contamination from the location where the exposure occurred, but as noted above, assumes the control arm has a similar underlying trend to the intervention arm.

## Poisson Regression Approaches

An approach that better addresses the limitations of the difference-in-difference design is a Poisson regression approach. Instead of using discrete periods before and after the exposure, this approach quantifies the exposure-outcome association by using a time series of outcome data. The regression model includes a variable for exposure period (unexposed, exposed). The model will also include a variable or variables capturing the time effects, and the model may include further variables capturing time-varying confounders. The inclusion of multiple variables means the model can estimate the exposure-outcome association independent of these other variables.

One example of this approach is a study of suicide rates in Queensland, Australia, in the early months of the COVID-19 pandemic (Leske et al., 2021). The study analyzed monthly suicide data from December 2015 to August 2020 with the exposure of interest being the February to August 2020 period. These data are available as ESM 4. After adjusted for underlying time trends, the authors found no evidence that the suicide rate in the exposure period had changed from pre-exposure trends (RR = 1.02, 95% 0.83–1.25). A second example of this design comes from a study of suicide rates in Spain where the exposure of interest was the onset of the Global Financial Crisis in the late 2000s (Lopez Bernal et al., 2013). The authors examined the monthly suicide rates before and after March 2008, the date they identified as the beginning of the crisis. They found that overall suicide rates decreased by 0.3% per month over the study period (RR = 0.997, 95% CI: 0.995–0.998) but that the onset of the crisis was associated with a level-change 8.0% increase in suicides (RR = 1.080, 95% CI 1.009–1.156).

The general form of the Poisson regression model incorporating time effects is:

$$\log\left(y_{i}\right)=\beta_{0}+\beta_{1}\times {\;\text{exposure}}_{i}+\beta_{2}{\times \text{time}}_{i}+\log\left({\text{population}}_{i}\right).$$

The exponential of the parameter $\beta_{1}$ is the rate ratio of interest, that is $e^{\beta_{1}}=\text{RR}$. This parameter represents a level-change in the outcome during the exposure period. Because this approach uses regression modelling, there is flexibility in how time effect can be modelled. Equation 5 illustrates the simplest approach, a linear time trend that assumes a constant effect of time on the outcome. More complex models allow for nonlinear time trends and can be fit to the data with polynomial terms (e.g., $\text{time}+{\text{time}}^{2}$), restricted cubic splines (Harrell, 2015), or fractional polynomials (Royston & Sauerbrei, 2008). It is also possible to account for short-term trends in the model. This can be done by imposing a structure on the data (e.g., indicator variables for months or quarters) or by estimating seasonality effects using Fourier terms (pairs of sine and cosine functions).

The approach taken in Equation 5 only considers the scenario where the exposure is associated with a level-change in the outcome. However, there is often interest in testing if the exposure is also associated with a slope change as well, that is, a difference in the rate of change over time in the exposed period compared with the unexposed period. This model is:

$$\log\left(y_{i}\right)=\beta_{0}+\beta_{1}\times {\text{exposure}}_{i}+\beta_{2}\times {\text{time}}_{i}+\beta_{3}\times \text{time after }{\text{exposure}}_{i}+\log\left({\text{population}}_{i}\right).$$

The key to setting this model up is to code a time after exposure variable that has values 0 in the unexposed periods and values 1,2,…,t in the exposed periods. The slope-change in the exposed period is therefore $e^{\beta_{2}+\beta_{3}}$. Lopez Bernal et al. (2017) discusses how a variety of different models can be fit that extend the hypotheses beyond level and slope changes (e.g., temporary change, slope-only change).

We illustrate the level and slope change model using a recent study assessing the impact of pesticide regulation on suicide in China (Yan et al., 2023). Regulations to restrict access to these substances were implemented over a number of years, leading to the authors to assess the impact of three tranches of regulation. For simplicity, we focus on describing the findings in relation to the initial change in regulation. Prior to regulation, suicide by this means was steady over time with no evidence of an increase or decrease (RR = 0.999 per month, 95% CI 0.998 to 1.001). In the month the regulation was implemented, there was no evidence of a level change (RR = 0.987, 95% CI 0.950–1.025), but there was evidence of a slope change. Specifically, each month after this – until the second set of regulation came into effect – suicide by this means declined by a factor of 0.993 per month (95% CI 0.991–0.994).

The Poisson regression approach can also account for other confounding factors. This could include other nonoverlapping interruptions or other factors changing over time. Thus, one of the advantages of this approach is its flexibility in that it can account for a variety of confounder variables and thus address a variety of sources of bias. Hawton et al. (2013) did this in a study evaluating the implementation of legislation to reduce pack sizes for a widely available substance.

The Poisson regression does have several limitations that should be considered when planning a study. Statistical power comes from both the number of time periods under observation and the number of events in each period. Studies with few periods and a low number of suicides per period will have lower power to detect effects than studies with many periods and high number of suicides per period. Second, there needs to be concordance between the period with which the exposure occurs and the outcome data (i.e., if the exposure changes from month to month, then monthly outcome data are required for analysis). Third, some exposures may take time to be become fully active, or the end of the exposure period may be poorly defined.

## Recommendations

Approaches that ignore underlying time trends and other confounders are at risk of misidentifying the association between population-level interventions and exposures and an outcome like suicide counts. The risk of misidentification is greatest for the pre–post and the difference-in-difference designs. This leads us to make the following recommendations:

1. The default position of anyone evaluating the association between a population-level intervention or exposure and an outcome like suicide should be a Poisson regression model that incorporates time effects. In the first instance, the models should test for a level change and a slope change, and if the term for the slope change is not statistically significant, then the model can be refit without this term. While these models are more complicated than techniques used for pre–post and difference-in-difference designs, the use of multivariable regression is now very common and these models are just another version of this. Time is essentially a confounder; accounting for this, where appropriate, will reduce bias in the exposure-outcome association, reduce the precision of exposure-outcome association (i.e. smaller confidence intervals), and increase power.
2. Aim to gather data on any other relevant factors that might be associated with the exposure and/or the outcome. These should be included in the analysis. Once the data have been analyzed, if these factors have no association with the outcome, then they can potentially be excluded. But even if these variables have no direct association with the outcome, they may still be adjusting the association between other variables and the outcome. Like time effects, including these variables will likely improve the precision of the other variables (including the exposure-outcome variable) and increase the power to detect an effect.
3. In situations where it is not possible to analyze population-level interventions or exposures using Poisson regression, the pre–post design and difference-in-difference designs could be employed but these should be seen as a fall-back position rather than a first choice. Researchers should be aware of the potential biases in these designs and note these as limitations.

In summary, despite the pre–post design’s widespread use in policy evaluation, as exemplified here with suicide prevention research, it has several significant limitations. These limitations mean that studies based on the pre–post design are at high risk of bias, reducing their usefulness for generating new knowledge. The difference-in-difference design offers an improvement on this, but Poisson regression approaches offer a better way of evaluating the impact of an intervention or an exposure. By incorporating more data into the analysis, and by using that data to account for potential time trends and confounding, researchers will have a greater chance of detecting the true impact of population-level interventions and exposures on suicide, thereby providing better advice to policymakers about how to reduce suicide rates.

## Electronic Supplementary Material

The electronic supplementary material is available with the online version of the article at https://doi.org/10.1027/0227-5910/a000961

**ESM 1.** Examples of data setup for each design, example code to undertake the analyses in Stata and R, and commentary aiding the interpretation of the results.

**ESM 2.** The Ladwig dataset as a csv file, illustrating the data structure for analyzing a pre--post design.

**ESM 3.** The Sinyor dataset as a csv file, illustrating the data structure for analysing a difference-in-difference design.

**ESM 4.** The Leske dataset as a csv file, illustrating the data structure for analyzing a Poisson regression model.

**ESM 5.** Stata code to undertake all analyses described here.

**ESM 6.** R code to undertake all analyses described here.
