# Supplementary material for: Evaluating Population-Level Interventions and Exposures for Suicide Prevention
Source: Crisis. 2024 May 21;46(1):50–5. doi: 10.1027/0227-5910/a000961 (PMC11783171; doi:10.1027/0227-5910/a000961)
Supplement: Supplementary file 1 [file cri_46_1_50_esm1.docx]

**Appendix**

1 Data 2

2 The pre-post design 2

3 The difference-in-difference design 5

4 Poisson regression approaches 8

4.1 The basic model 9

4.2 Accounting for non-linear long term time trends 10

4.3 Accounting for short-term seasonality trends 14

4.4 Combining long and short-term time trends 15

4.5 Accounting for level and slope changes 16

5 References 18

# Data

All data used in this appendix are publicly available and have been uploaded alongside this document as an electronic supplementary appendix. The files are Ladwig.csv (for the pre-post design), Sinyor.csv (for the difference-in-difference design) and Leske.csv (for Poisson regression incorporating time effects). We give illustrative code for how to use this data in Stata. R code, doing much the same thing, has also been uploaded. These files are “Stata_examples.do” and “R_examples.do”.

All three datasets can be imported and labelled in Stata using the following commands.

**// Pre-post design**

import delimited "Ladwig.csv", varnames(1) clear

label var suicides "Number of suicides"

label var period "Pre or post exposure period"

label var time "Observation time (days)"

label define period 0 "Pre-exposure" 1 "Post-exposure"

label values period period

label data "Ladwig et al., 2012. Type -notes- for DOI"

notes: Original study available from https://doi.org/10.1016/j.jad.2011.09.044

save "Ladwig", replace

**// Difference-in-difference design**

import delimited "Sinyor.csv", varnames(1) clear

label var suicides "Number of suicides"

label var period "Pre or post exposure period"

label var year "Year"

label var arm "Experimental or Control Arm"

label var time "Observation time (in years)"

label define period 0 "Pre-exposure" 1 "Post-exposure"

label values period period

label define arm 0 "Control" 1 "Experimental"

label values arm arm

label data "Sinyor et al. 2017, type -notes- for link to study"

notes: Original study available from: https://bmjopen.bmj.com/content/7/5/e015299

order year arm period suicides time

save "Sinyor", replace

**// Poisson regression with time effects**

import delimited "Leske.csv", varnames(1) clear

format date %tm

label var date "Year and month"

label var time "Time"

label var period "Pre or post exposure period"

label var suicides "Number of suicides"

label var pop "Population size"

label define period 0 "Pre-exposure" 1 "Post-exposure"

label values period period

label data "Leske et al. 2021, type -notes- for link to study"

notes: Original study available from: https://doi.org/10.1016/S2215-0366(20)30435-1

save "Leske", replace

# The pre-post design

An example of a single-arm pre-post design is from a study of suicides on the German rail network following the suicide of a well-known footballer by the same method (Ladwig et al., 2012). In that study, the number of suicides in the 28 days following the footballer’s death were compared to the number of suicides in the preceding 28 days. The data can be set up for analysis with three variables (suicides, period, time) in Stata as follows:

The variable suicides refers to the number of suicides in each period. Period is coded 0 in the pre-exposure period and 1 in the exposure period. Time refers the observation time of each period. Using this data, the rate ratio, $RR$, for a single-arm pre-post design can be calculated with the following command using equation 1 in the main document.

This gives a rate of 121 / 28 = 4.32 suicides per day in the exposed group and 53 / 28 = 1.89 per day in the unexposed group. The $RR$ is therefore 4.32 / 1.89 = 2.28, interpreted as the number of suicides being 2.28 times higher in the exposed period compared to the unexposed period. The 95% confidence interval for this estimate is 1.64 to 3.22.

As noted in the main document, the $RR$ can also be calculated using Poisson regression where the number of suicides is the outcome, period is entered as an indicator variable and time is included as an offset term. This command is:

These estimates are all on the log scale. The rates in each period can be calculated using post-estimation commands that present the results on the exponential scale. The rate in the unexposed period is:

and the rate in the exposed period is:

Finally, the $RR$ is:

While the $RR$ calculated using Poisson regression is the same as calculating the $RR$ directly using equation (1), the confidence intervals differ slightly. The direct calculations use a formula referred to as the exact method whereas Poisson regression uses a normal approximation based on the z distribution. However, differences between the two methods are generally small and unimportant unless the number of outcome events is low.

# The difference-in-difference design

Use of the difference-in-difference design in suicide prevention is illustrated by study of jumping suicides at the Bloor Street Viaduct (Sinyor et al., 2017). The study compared the number of jumping suicides before and after installation of a safety barrier at the viaduct to jumping suicides from other bridges in Toronto over the same time periods.

When the outcome is a count variable like the number of suicides, the difference-in-difference design compares the rate of change in an experimental arm to the control arm. The rate of change is defined as the change in the number of events from the unexposed period to the exposed period. Formally, this is estimated by $RR$, which is defined as the ratio of ${RR}_{e}$ to ${RR}_{c}$, where $RR_{e}$ is the pre-post rate ratio in the experimental arm and $RR_{c}$ is the pre-post rate ratio in the control arm. This can be estimated using the following Poisson regression model:

$$\begin{aligned} \log\left( y_{i} \right)= \beta_{0}+ \beta_{1}\times\text{experimental arm}_{i}+ \beta_{2}\times\text{exposure}_{i}+ \\ \beta_{3}\times\text{experimental arm}_{i}\times\text{exposure}_{i}+\log\left( \text{tim}\text{e}_{\text{i}} \right).\# \end{aligned}$$

where $y_{i}$ is the number of suicides in $\text{time}_{i}$; $\text{experimental }\text{arm}_{i}\text{ }$is a binary coded variable that is equal to 1 if the observation is from the experimental arm and 0 if it is from the control arm; $\text{exposure}_{i}$ is a binary coded variable coded 1 is the observation from the exposed period and 0 if it is from the unexposed period; and $\text{experimental }\text{arm}_{i}\times\text{exposure}_{i}$ is the product of these two variables.

Using this model, the rates in the four groups that define the design can be calculated by substituting different values into the equation. Focusing on the experimental arm, in the exposed period, $\text{experimental arm}=1$ and $\text{exposure}=1$, therefore, the equation can be re-written as follows:

$\begin{aligned} \log\left( y_{i} \right)= \beta_{0}+ \beta_{1}\times1+ \beta_{2}\times1+ \beta_{3}\times1\times1 \\ \# \end{aligned}$This gives a rate of $exp(\beta_{0}+ \beta_{1}+ \beta_{2}+ \beta_{3})$. Similarly, in the unexposed period, $\text{experimental arm}=1$ and $\text{exposure}=0$, therefore the is $exp(\beta_{0}+ \beta_{1})$. Focusing on the control arm, in the exposed period, $\text{experimental arm}=0$ and $\text{exposure}=1$, so the rate is $exp(\beta_{0}+ \beta_{2})$ while in the unexposed period, both $\text{experimental arm}$ and $\text{exposure}$ equal 0, meaning the rate is $exp(\beta_{0})$. Using these equations, it can be shown that $RR$ is equal to $exp(\beta_{3})$.

$$RR= \frac{RR_{e}}{RR_{c}}=exp\left( \frac{\frac{\beta_{0}+ \beta_{1}+ \beta_{2}+ \beta_{3}}{\beta_{0}+ \beta_{1}}}{\frac{\beta_{0}+ \beta_{2}}{\beta_{0}}} \right) =exp(\beta_{3})$$

Returning to the study of the Bloor Street Viaduct (Sinyor et al., 2017), the first 5 observations in the data are as follows:

The variable year refers to the year the data is from. It is not needed for analysis but is helpful for contextualising the data. The variable arm refers study arm, that is control or experimental arm. It is coded 0 for the control arm and 1 for the experimental arm. The variable period represents the unexposed or exposed periods and is coded 0 in the unexposed period and 1 exposed period. The variable suicides refers to the number of suicides in each arm and period. Time refers to the number of years over which the data is gathered, and while not needed for analysis here because time is constant, we include it to show how to deal with time when it is not constant.

For demonstration, we first calculate the suicide rates and rate ratios directly in the experimental and control conditions.

In the experimental arm, the rate in the unexposed period it is 105 / 11 = 9.55 suicides per year while in the exposed period it is 1 / 11 = 0.09 suicides per year. ${RR}_{e}$ is therefore 9.55 / 0.09 = 0.0095.

Similarly, in the control arm, the rates are calculated as follows:

which give ${RR}_{c}$ = 10.1 / 11.0 = 1.09. As noted in equation (3) in the main document, the $RR$ is therefore 0.0095 / 1.09 = 0.0087. These values can all be calculated with Poisson regression.

These are coefficients and therefore on the log scale. These coefficients can be converted to rates in each arm and period using the following postestimation commands:

And $RR$ is calculated with the command:

Which is the same as that calculated directly from the rates.

# Poisson regression approaches

An example of a study that Poisson regression that incorporated time effects in suicide prevention is a study by Leske et al. (Leske et al., 2021). In that study, the authors were interested in any change in suspected suicide rates in the early stages of the Covid-19 pandemic. They collected monthly data from January 2015 to August 2020, with the exposure defined as the period from February 2020 onwards. Their full dataset is available in a supplementary appendix with the original article. We use a subset of the data, with the first five observations displayed below.

The data consist of variables for month and year, time (coded 1, 2, …, 68), period (coded 0 prior to February 2020 and 1 thereafter), suicides (the number of suicides per month) and pop (the estimated population size). We demonstrate how to fit a Poisson regression model using a variety of different ways to account for long-term and short-term time trends.

## The basic model

The simplest model is one where the association of time on the number of suicides is assumed to be linear (constant). The model is fit using the -glm- commands in Stata. As before, the key variable in the model is an indicator variable for period, but the command also includes the population size as an offset term -exposure(pop)- and the scaling parameter -scale(x2)- to account for overdispersion (where the variance is greater than the mean). The full command is as follows:

As in our earlier examples, the rate ratio of interest, $RR$, is the exponential of the coefficient for the post-exposure period. This can be calculated using a postestimation command and in this case is 0.94 (95% CI 0.82 to 1.06) which is not significantly different from the null value of 1.00 (p = 0.318).

## Accounting for non-linear long term time trends

The assumption that the association between time and suicides is linear can be explored by fitting a range of different models that allow for a non-linear association. One option is to use polynomial terms (e.g., $x+x^{2}$, where $x$ = time). This can be done as follows:

This gives the following rate ratio for the exposure period:

For a variety of reason, polynomial terms like this are discouraged in health research, principally because they can lead to predictions that are biologically implausible, i.e. they do not normally occur in social or environmental processes (Royston & Sauerbrei, 2008). One alternative is to use restricted cubic splines (Harrell, 2015) which fit a continuous line through the variable of interest that varies in shape between a set of knots. The knots can be defined in a variety of ways, but the default is to place 5 knots at equally spaced intervals. The resulting splines are linear before the first knot, non-linear between the adjacent knots and linear after the last knot. This can be done as follows:

As in the previous models, the key parameter of interest is the effect of the exposure period. Once again, this is calculated with the postestimation command:

An alternative to fitting restricted cubic splines is to fit fractional polynomials terms. Fractional polynomials are similar to polynomials but allow a much greater range of shapes. In the default settings, all 44 combinations of polynomials with values -2, -1, -0.5, 0, 0.5, 1, 2, 3, where $0=log(x)$ and all other powers are $x^{-2},x^{-1}$ etc, are fit to the data with the best fitting selected for the final model. This is done using up to two transformations of $x$, so for instance, one possible polynomial is $x^{2}+log(x)$. If two powers are the same, then the convention is that the variable includes $log(x)$, i.e, powers of 2, 2 becomes $x^{2}+x^{2}*log(x)$. Thus, the polynomial $x+x^{2}$ that we used for above is simply one of all possible combinations of polynomials that are available under the default settings. Fractional polynomials for time can be fit to the data as follows:

In this case, the algorithm has selected the powers 3, 3, meaning the transformation for time is $t^{3}+t^{3}*log(t)$, where $t$ is the variable time. (Note that in this example, a model with this transformation offers no improvement over a model with just a linear term for time, p = 0.193 from the top box, or even one with time omitted altogether, p = 0.464. Like the previous examples, this suggests no association in this data between the number of suicides per month and time.) Returning to the issue of the effect of the exposure, this is calculated as follows:

## Accounting for short-term seasonality trends

In addition to long-term time trends, it is possible that short-term seasonality effects may be in play. This can be tested by using Fourier terms – a set of sine and cosine pairs that are included in the model. This is done by generating the following variables:

and then including these variables in the model.

Once again, the RR is calculated with this command:

## Combining long and short-term time trends

The long-term and short-term trends can be combined into a single model. Using the fractional polynomials as an example,

leading to the following estimate of the RR:

Alternatively, the long-terms trends can be combined with the short-term trends using restricted cubic splines as follows:

## Accounting for level and slope changes

Finally, the examples above all illustrate how to fit models when the hypothesis is that there is a level change in the number of suicides during the exposure period. An alternative hypothesis is that there is a level and slope change. To estimate the slope change, we first generate a new variable code 0 in the pre-exposure period and $1, 2, \ldots, t$ in the exposure period. This can be done as follows:

We then fit a model with this term included.

The estimate of the level change is:

And the estimate of the slope change is:

These findings suggest a non-significant 20.6% increase in the number of suicides in the month when the exposure period began followed 6.3% reduction in suicides per month after that.

# References

Harrell, F. E. (2015). *Regression modeling strategies: with applications to linear models, logistic and ordinal regression, and survival analysis*. Springer.

Ladwig, K.-H., Kunrath, S., Lukaschek, K., & Baumert, J. (2012). The railway suicide death of a famous German football player: impact on the subsequent frequency of railway suicide acts in Germany. *Journal of affective disorders*, *136*(1-2), 194-198.

Leske, S., Kõlves, K., Crompton, D., Arensman, E., & De Leo, D. (2021). Real-time suicide mortality data from police reports in Queensland, Australia, during the COVID-19 pandemic: an interrupted time-series analysis. *The Lancet Psychiatry*, *8*(1), 58-63.

Royston, P., & Sauerbrei, W. (2008). *Multivariable model-building: a pragmatic approach to regression anaylsis based on fractional polynomials for modelling continuous variables*. John Wiley & Sons.

Sinyor, M., Schaffer, A., Redelmeier, D. A., Kiss, A., Nishikawa, Y., Cheung, A. H., Levitt, A. J., & Pirkis, J. (2017). Did the suicide barrier work after all? Revisiting the Bloor Viaduct natural experiment and its impact on suicide rates in Toronto. *BMJ Open*, *7*(5), e015299. <https://doi.org/10.1136/bmjopen-2016-015299>
